# Personalisation and profiling using algorithms and not-so-popular Colombian music: goal-directed mechanisms in music emotion recognition

**DOI:** 10.1140/epjds/s13688-025-00595-1

**Published:** 2025-11-13

**Authors:** Juan Sebastián Gómez-Cañón, Thomas Magnus Lennie, Tuomas Eerola, Pablo Aragón, Estefanía Cano, Perfecto Herrera, Emilia Gómez

**Affiliations:** 1 Music Technology Group, Universitat Pompeu Fabra, Barcelona, Spain; 2 School of Medicine, Stanford University, Palo Alto, USA; 3 Department of Music, Durham University, Durham, UK; 4 American University in Bulgaria, Blagoevgrad, Bulgaria; 5 Universitat Pompeu Fabra, Barcelona, Spain; 6 Songquito, Erlangen, Germany; 7 Joint Research Centre, EU Commission, Seville, Spain

**Keywords:** Music emotion recognition, Polarisation, Profiling, Colombia, Personalisation

## Abstract

This work investigates how personalised Music Emotion Recognition (MER) systems may lead to sensitive profiling when applied to musically induced emotions in politically charged contexts. We focus on traditional Colombian music with explicit political content, including (1) vallenatos and social songs aligned with the left-wing guerrilla Fuerzas Armadas Revolucionarias de Colombia (FARC), and (2) corridos linked to sympathisers of the right-wing paramilitary group Autodefensas Unidas de Colombia (AUC). Using data from 49 participants with diverse political leanings, we train personalised machine learning models to predict induced emotional responses – particularly negative emotions. Our findings reveal that political identity plays a significant role in shaping emotional experiences of music with explicit political content, and that emotion recognition models can capture this variation to a certain extent. These results raise critical concerns about the potential misuse of emotion recognition technologies. What is often framed as a tool for wellbeing and emotional regulation could, in politically sensitive contexts, be repurposed for user profiling. This work highlights the ethical risks of deploying AI-driven emotion analysis without safeguards, particularly among populations that are politically or socially vulnerable. We argue that subjective emotional responses may constitute sensitive personal data, and that failing to account for their sociopolitical context could amplify harm and exclusion.

**Supplementary Information:**

The online version contains supplementary material available at 10.1140/epjds/s13688-025-00595-1.

## Introduction

Emotion is one of the main reasons people engage with music (Juslin et al. [43]), yet our understanding of musically induced emotional experiences remains incomplete. Recent models of music-evoked emotions (Cespedes-Guevara [15], Lennie and Eerola [50]) increasingly incorporate individual and contextual factors, drawing from contemporary affective and cognitive science (Eder and Hommel [24], Frijda [29], Moors [55], Russell [67], Schiller [72]). Among these factors, goal-directed mechanisms play a key role by prioritising emotionally relevant stimuli according to personal values, needs, and goals. This perspective reinforces the idea that music’s emotional impact is deeply personal and important in contexts where individual emotional responses intersect with sociopolitical identities.

Music Emotion Recognition (MER) systems, powered by machine learning, attempt to predict the emotions perceived or induced by music in listeners (Yang and Chen [87]). These predictions underpin psychology-informed recommender systems (Schedl et al. [70,71]), which aim to enhance the user experience by aligning music suggestions with emotional states. However, building effective MER models requires emotional “ground truth” data – a term that refers to the “real” or “true” information that the algorithm attempts to predict (Schuller [74]). To address this, recent efforts have focused on gathering richer, listener-specific annotations, including demographic, cultural, and situational information (Barthet et al. [10], Gómez-Cañón et al. [32], Schedl et al. [70]). This has enabled the development of personalised models (Chen et al. [18], Gómez-Cañón et al. [33], Su and Fung [79], Yang et al. [88]), which in turn support more precise, emotionally relevant music recommendations.

While these advancements offer new possibilities for emotional wellbeing and user-centred design, they also raise pressing ethical concerns – especially when personalised recommendations intersect with vulnerable populations or politically sensitive content. Personalised systems influence user behaviour and autonomy (Calvo et al. [13]), and the opacity of their recommendation criteria has implications for bias, polarisation, and democratic discourse (Grisse [36]). A recent European Commission report warns against the potential manipulation inherent in “toying with emotions combined with personalisation” (Lupiáñez-Villanueva et al. [51]). Music, as a functional and persuasive medium, has long been used to convey identity, ideology, and power (Brown and Volgsten [12], Townsend [82]). In emotionally adaptive systems, a user’s political or social identity might be inferred from emotionally charged musical preferences – especially if recommendations are made public, such as in shared playlists. This possibility highlights the need to design systems not only for engagement or entertainment, but also with transparency, accountability, and inclusion in mind. As Townsend writes, “*the ability to control the music we hear and play is such a powerful tool that it has always been exploited by those in positions of political or religious power, and equally by those seeking change or revolution*” (Townsend [82]).

In this study, we explore how personalised MER systems may encode or reveal political identities through the emotional profiling of traditional Colombian music with explicit ideological content. We argue that emotional data – often framed as benign or neutral – can, in politically sensitive contexts, become a vector for vulnerability and misuse. Colombia’s complex history of civil conflict, paramilitarism, and polarised media makes it a salient case for examining how algorithmic music systems may reinforce vulnerabilities in politically divided societies. Our work aims to surface the ethical implications of emotion-aware systems and contribute to ongoing discussions about the responsible use of data and AI for wellbeing.

### Aims

This study extends the work by Gómez-Cañón et al. [35] and Lennie and Eerola [50] by examining whether personalised Music Emotion Recognition (MER) algorithms can effectively predict music that elicits “negative” emotional responses in listeners. We use the term “negative” carefully, acknowledging the subjectivity involved in emotional interpretation. Nonetheless, the use of personalised emotion-based recommendations – already standard in streaming platforms – raises important questions about their psychological and sociopolitical impact, particularly when music is politically charged.

Music has long served as a tool for group identity and ideological expression. For example, work songs and community choirs foster shared purpose, while political music can mobilise and polarise (Pedelty and Keefe [62], Townsend [82]). If recommender systems begin to leverage listeners’ emotional profiles, it is critical to assess not only their benefits (e.g., mood regulation), but also the risks, such as reinforcing ideological bias or triggering harmful psychological states (Ahonen [3], Kennaway [46], Short and Dingle [76], Silverman et al. [77]).

Other troubling findings have been discovered in personalised media environments – such as those created by recommender systems – can increase emotional susceptibility, compliance, and polarisation (O’Neil [59], Ziv [92], Zuboff [93]). A massive experiment showed empirical evidence of emotional contagion through the exposure of users to posts from social media expressing specific emotions (Kramer et al. [48]). The data scandal with Cambridge Analytica demonstrated how emotion profiling can potentially persuade users, polarise opinions, and affect decision-making processes by promoting/manipulating individualised emotional stimuli (Davies [22]). Such systems risk creating “filter bubbles” and “echo chambers” (Pariser [61], Sunstein [80]), limiting users’ exposure to diverse viewpoints. In contrast, algorithmic design can also foster depolarisation (Garimella et al. [30], Stray [78]).

We explore these tensions in a politically sensitive setting: the Colombian presidential elections. During this period, citizens displayed heightened emotional and political engagement. We leverage this context to design an experiment combining: (1) music and lyrics as emotionally salient stimuli; and (2) personalised MER algorithms predicting emotional responses to those stimuli. Our goal is to assess whether these models can learn patterns tied to users’ political orientation and emotional sensitivity.

Colombia’s conflict history, involving politically motivated violence and stark ideological divisions, provides a uniquely rich context (Chomsky [20], Fals Borda [28], Mahoney [52]). Multiple forms of structural inequalities have fueled the rise of politically motivated illegal armed groups in Colombia, including the left-wing Fuerzas Armadas Revolucionarias de Colombia (FARC) and right-wing Autodefensas Unidas de Colombia (AUC). The “left-wing” guerrilla group FARC is associated with *vallenatos*^1^ and *canción social* that support their political ideology. Similarly, the *corridos prohibidos* – ballads often associated with the 20th century Mexican revolution and later with the narcotics trade (Barbosa Caro and Suavita [9], Villalobos and Ramírez-Pimienta [84]) – have been used by several sympathisers of the “right-wing” paramilitaries AUC. While not widely popular, their lyrics express clear partisan ideologies that are well understood by Colombian listeners.

By analysing listener affective reactions to these songs and the predictions made by personalised MER models, we aim to understand how such systems may shape political perception and emotional experience. This study ultimately seeks to inform ethical and responsible design practices for emotionally aware recommendation technologies, especially in contexts involving vulnerable populations and polarised media.

In this study, we formulate the following research questions:

1. Do an individuals’ political values influence emotional episodes induced by music in terms of core affect^2^?
2. Can a MER algorithm for induced emotions be biased towards particular political values with respect to music with polarising lyrics?

### Hypotheses

We begin by defining key terms central to our study. Goal-directed mechanisms refer to cognitive processes shaped by personal goals, such as needs, values, or beliefs (Moors [56]). Two important components are: 1) goal relevance – whether a stimulus matters to an individual’s goals (“Does X matter?”), and 2) goal congruence – whether a stimulus supports or hinders those goals (“Does X help or hurt?”) (Kuppens et al. [49]). Core affect refers to fundamental affective states, typically described along the dimensions of pleasantness and arousal (Russell [67]). These concepts frame our investigation of how politically charged music – when aligned or misaligned with listeners’ goals – can influence core affect. This has direct implications for Music Emotion Recognition (MER) systems, which increasingly rely on personal and contextual data. Despite this trend, MER has not been evaluated within a fully personalised, goal-sensitive framework (Gómez-Cañón et al. [32], Hu et al. [40]).

Our approach uses a goal-directed perspective to guide MER personalisation, enhancing its ability to adapt to individual preferences and contextual factors. The Colombian presidential election of 2022 provided a natural manipulation of goal relevance, as political identities become highly salient. We expect that music congruent or incongruent with listeners’ political views will influence emotional responses, especially under goal-incongruent conditions.

We test two hypotheses:

1. Politically sensitive music stimuli that agrees/disagrees (goal congruence) with participants political stance (goal relevance) will lead to differentiated core affect annotations.
2. Personalised algorithms will effectively bias towards specific affective quadrants – distinct emotion regions defined by the dimensions of valence (positive vs. negative) and arousal (high vs. low) – depending on the political stance of listeners. That is, personalised algorithms reflect listeners’ political views and can thus be used to make tailored emotion-based recommendations.

To verify these hypotheses, we designed a study in which we use music with politically charged lyrics and train MER models using different personalisation strategies. We systematically evaluate the annotations of listeners with different political leanings and evaluate if MER models can reflect their political views.

## Methodology

### Experimental setup

The experiment was held in an online platform developed using React and a Flask backend. It was developed in English (for design purposes) and translated to Spanish (for the participants).^3^ The experiment was first piloted with 5 Colombian nationals. Participants were collected through social media outlets (Twitter and Instagram) targeting Colombian nationals over the course of 2 months (June-July) in the run up to the 2022 presidential elections and after the first round of elections. We also invited researchers from the following Colombian universities: Universidad Central, Universidad de Antioquia, Universidad de los Andes, and Universidad Nacional. Participants were incentivised through a prize draw for one of four €100 gift vouchers. The experiment took an average of 30-40 minutes to complete. We summarise our experimental procedure as follows:

1. Participants agreed to the consent form validated by the ethics committee from the Universitat Pompeu Fabra (in conformity with GDPR and Colombian data protection laws) – stating that they are Colombians and are between 18 and 65 years old.
2. Participants provided general demographic variables: age, gender, native language, and musical self-identification.
3. A short attention check followed: three sounds in randomly assigned order were played and each participant should select the one with lowest sound level. If the participant was unable to complete this step, they were informed that their device did not have the audio fidelity to continue with the experiment.
4. Participants received an explanation on how to complete the annotations: a comparison between perceived and induced emotions in music and a description of the annotation interface (see section on annotation gathering).
5. Participants listened to and annotated the music: (a) each participant was randomly assigned a different personalisation strategy: a model trained with acoustic features (*ACO*), a model trained with features from the lyrics (*LYR*), a multi-modal model that takes into account both lyrics and acoustic features (*MIX*), and a pseudo-random baseline that presented music to perform an annotation consistency check (*RAND*) ;^4^ (b) initially, all participants annotated the same 6 tracks for the first iteration (2 FARC-songs; 2 AUC-songs; 2 songs without lyrics that were randomly selected) to train personalised models (see section Personalisation); (c) based on these initial annotations, the personalised model was retrained and queried to select a new batch of 6 tracks for the participant to annotate; and (d) the personalised model was then refined using the remaining tracks presented across three additional iterations of 6 tracks each (for a total of four personalised iterations, including step c). In total, each participant annotated 30 tracks (6 initial + 4 × 6 personalised), following past literature on personalisation (Chen et al. [19], Gómez-Cañón et al. [33]).
6. Finally, participants completed three questionnaires on the political opinion – Right-Wing Authoritarianism scale (RWA), the Social Dominance Orientation scale (SDO), and a Colombian specific political questionnaire made for the purpose of this study. We use this information to create groups of political opinions and analyse the resulting models and their output.

### Scales

*Musical self-identification* was measured by the Ollen Musical Sophistication Index (OMSI). This single item scale provides estimates of the psychometric measures used to determine the membership of the category “musician” (Ollen [58]). The OMSI musician rank item is concerned with the individual’s self-assessed level of musical identity as opposed to an item related to musical expertise (Zhang and Schubert [91]).

The *right-wing authoritarianism* (RWA) scale, originally developed by Altemeyer [6], measures social attributes, such as the degree to which people defer to established authorities, show aggression toward out-groups when authorities sanction that aggression, and support traditional values endorsed by authorities (Saunders and Ngo [69]), racism and sexism (Zakrisson [90]). We used the short 15-item version of the RWA (Zakrisson [90]) which uses less extreme and more modern language, and makes less reference to specific groups (e.g., women). Higher scores indicate stronger authoritarian attitudes; lower scores indicate anti-authoritarian attitudes and weaker endorsement of traditionalist norms.

*Social dominance orientation* (SDO) refers to the extent to which a person desires that one’s in-group dominate and be superior to out-groups. SDO is considered to measure social and political attitude orientation toward inter-group relations, reflecting whether one generally prefers such relations to be equal, versus hierarchical, that is, ordered along a superior-inferior dimension (Pratto et al. [63]). We used the updated SDO7 (Ho et al. [37]) which is significantly shorter (8 items) and uses more modern references compared to the original iterations. Higher scores indicate a stronger preference for hierarchical intergroup relations; lower scores indicate stronger preferences for egalitarianism and group equality.

Both the RWA and the SDO scales are said to capture two different dimensions of political opinions (Asbrock et al. [7]). This two-dimensional interpretation offers a substantial amount more nuance to our interpretation of Colombian voters. Scales can be seen to capture different aspects of political values, goals, and motivations – we used them to group the participants in our experiment. The scales show a reasonable degree of correlation in capturing left/right political view points. Both scales were measured on a 5-point Likert-like scale (original scales used a 1–9 rating). However, for ease of use with mobile phones, we shortened the scale for compatibility. Importantly, all political scales were measured on the same scale to allow for equivalence. Higher ratings on both the SDO and the RWA suggest a stronger right-wing political ideology.

The *Colombian specific political questionnaire* was developed during the pilot section of the experiment through participant suggestions for extensions of the RWA or SDO scales. Although both scales have been used in cross-cultural contexts (Duckitt et al. [23]), including other collectivist cultures in South America (Cantal et al. [14]), neither has been validated in a Colombian setting to our knowledge. Specifically, candidates’ proposals addressed the Colombian political climate: political nuances are idiosyncratic to the electoral time frame. We concluded that such a measure would be highly beneficial in assessing Colombian political dimensions and countering the limitations in the RWA and SDO. We chose nine items to represent several key components of the Colombian elections – three proposals from each candidate that identified the political “center”, “right”, and “left” were rated by each participant, also on a 5-point scale (see full scales in supplementary material A.)

### Music selection

We refer the reader to studies by Quishpe [64], Barbosa Caro and Suavita [9], and Katz-Rosene [45] with respect to historical, functional, and lyrical analysis of the two types of music used: (1) FARC-songs (mainly in the style of *vallenato* and *canción social*) and (2) AUC-songs (in the style of *corridos*). These musical styles are part of traditional Colombian (and Latin-American) music, but they have distinctive sonorities, structures, and instrumentation. It must be noted that music with politically motivated lyrics from both types has incorporated other similar styles of music as well (e.g., hip-hop and rock), but this study only considers this reduced range of styles. Additionally, FARC-songs have been typically created by active members of the guerrilla as a mechanism of identity confirmation and propaganda (Quishpe [64]), while AUC songs have typically been produced by sympathisers of the paramilitaries as promotion to their deeds and in open criticism to the FARC and left-wing politicians (Barbosa Caro and Suavita [9]). Crucially, the functionality of the music and the target listener can be seen as different.

We remark that humans frequently listen to music *without* feeling any emotion at all (Juslin [42], Kivy [47]), but music *might* trigger mechanisms such as episodic memories for particular individuals (Eerola [25], Juslin [41]). However, the potential induction of emotions from the music in this study is based mainly on the semantic content of the lyrics – inducing different emotions to listeners with different political views. In Gómez-Cañón et al. [35], we exclusively evaluated acoustic features from the music – in this study, we extended the analysis to features in the lyrics as analysed in natural language processing and topic modelling (see the personalisation section for the description of computational models). However, acoustic features are useful for providing a content-based contrast among the different styles of music: (1) FARC-songs typically use less instruments and might include only voice and guitar, and (2) AUC-songs are more heavily orchestrated with faster tempo. Namely, the machine learning models should be able to differentiate between the types of music – the interesting element is to attempt to understand which users will provide problematic labels (i.e., music that induces subjectively negative emotions) that can bias the algorithm towards a particular class.

We started from a pool of 100 songs per music type and first evaluated their lyrics for political content. From these, we selected 50 excerpts with lyrics (30 seconds each) per type. For each excerpt, we extracted 260 emotionally relevant acoustic features (mean and standard deviation of 65 low-level music descriptors and their first-order derivatives) from segments of 1 second (Aljanaki et al. [5]), with 50% overlap, and standardise across features – using the IS13 ComParE feature set (Weninger et al. [86]) and OpenSMILE toolbox (Eyben et al. [27]). We processed the tracks using AudiosourceRe DeMIX software to extract versions without lyrics 5 – we obtained a total set of 150 excerpts (50 FARC-songs, 50 AUC-songs, 25 FARC-songs without lyrics, and 25 AUC-songs without lyrics). Importantly, each excerpt with lyrics was taken from a different song, thereby avoiding direct data leakage across fragments. For the set without lyrics, we selected 25 excerpts per group based on the quality of vocal suppression, ensuring control for acoustic artifacts. Each excerpt was normalised for loudness following the ITU-R BS.1770-4 recommendation using the pyloudnorm package.^6^

### Annotation gathering

**Figure 1:**
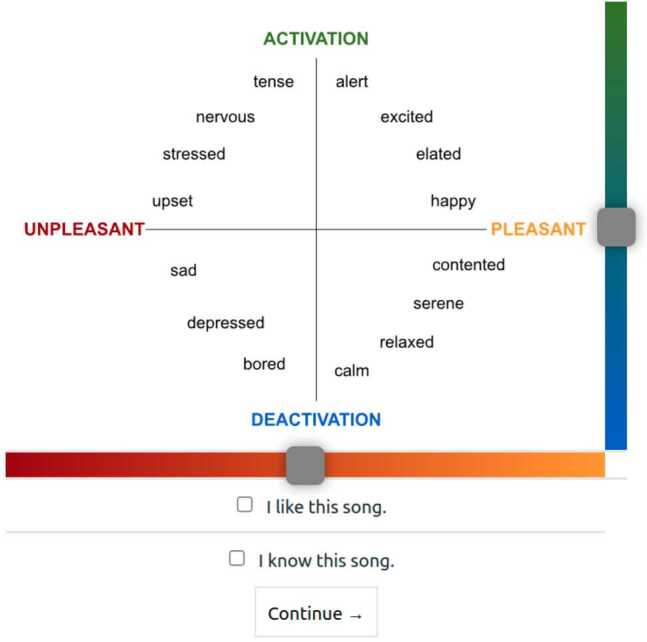
Annotation interface for the experiment

We use a discrete version of emotion based on Russell’s circumplex model (Russell [66]) and recent work on MER (Gómez-Cañón et al. [32], Panda et al. [60]), which conceptualises emotions into two core affects (arousal and valence) and four distinct categories/quadrants of emotion: $Q_{1}$ (positive valence and arousal), $Q_{2}$ (positive arousal and negative valence), $Q_{3}$ (negative valence and arousal), $Q_{4}$ (negative arousal and positive valence). Figure 1 shows the annotation interface: $Q_{1}$ refers to emotions such as happy and excited, alert; $Q_{2}$ refers to emotions as tension and anger; $Q_{3}$ refers to emotions as sadness and boredom; $Q_{4}$ refers to emotions as calmness, serenity. To refer to arousal, we used the words activation/deactivation (*activación/desactivación*). To refer to valence, we used pleasant/unpleasant (*positivo/negativo*). Annotations of arousal and valence were made on continuous sliders ranging from −100–100. We use continuous scale values to analyse annotations but use the discrete classes to train our machine learning models. We also collected each participant’s preference and familiarity for the musical excerpts through the check boxes “*I know this song*” and “*I like this song*” (see Fig. 1).

### Personalisation

We use the “machine consensus” MER personalisation strategy presented by Gómez-Cañón et al. [33]: consensus entropy for active learning. This strategy uses a committee of classifiers to analyse their output agreement and queries each user for instances with the highest uncertainty. We use a classification strategy since personalisation is achieved through the measure of entropy – a measure of uncertainty based on the predicted probabilities that an music excerpt belongs to a given class. Each participant receives a committee of classifiers: 5 independent extreme gradient boosting models (Chen and Guestrin [17]) and 5 logistic regression models optimised with stochastic gradient descent (Bottou [11]). Each one of these models had previously been pretrained on separate cross-validation splits of the DEAM dataset, the benchmark dataset for MER (Aljanaki et al. [5]). In order to select uncertain data to be labelled, classifiers predict the output probabilities for the pool of excerpts. We then perform the consensus entropy strategy by analysing the disagreement across classifiers. For example, full disagreement from a committee of four classifiers results when each one predicts a different class/quadrant with 100% probability. This yields average probabilities per quadrant $p_{avg} = \{Q_{1}: 0.25, Q_{2}: 0.25, Q_{3}: 0.25, Q_{4}: 0.25\}$ and high inter-class entropy/uncertainty of 1.386. Following Gómez-Cañón et al. [34], we balanced the instances with respect to the quadrants for each epoch: (1) prior to the calculation of entropy, we split the probabilities $p_{avg}$ into four matrices corresponding to the instances with higher probability of belonging to each quadrant, (2) we calculate entropy independently for each matrix (four quadrant probabilities × 150 instances), and (3) we select instances with highest entropy from each matrix. Thus, we alleviated the issue of imbalanced classes for each retraining iteration, since the instances selected for query are more likely to belong to each of the quadrants. In the case that the probabilities do not favour a particular quadrant (i.e., models are biased towards particular classes), we simply select the instances with highest entropy from the initial matrix. Excerpts with highest uncertainty are then queried to each participant to be annotated. Initially, we pseudo-randomly draw 2 excerpts from each type of music (6 excerpts for the first annotation iteration), retrain our classifiers with the annotations provided by each user, identify the excerpts to be annotated for the next iteration, and present the new batch of music to be annotated. Given the low amount of available music, we perform only five iterations for a total of 30 annotations per user – past research has shown that only 20-30 annotations are needed to achieve personalisation (Chen et al. [19], Su and Fung [79]).

In order to extract information from lyrics, we used a standard topic modelling approach (Manning and Schütze [53]) – an unsupervised method that detects word patterns within different texts and attempts to cluster documents (i.e., lyrics) into a particular amount of topics. The classical bag-of-words approach was implemented by: (1) calculating the frequency of words from each lyric (i.e., term frequency-inverse document frequency); (2) testing different algorithms to obtain a numeric representation of the likelihood of each lyric to belong to topic *t*; (3) using the extracted text features as input to a logistic regression classifier that is subsequently trained with the annotations of each participant. However, short texts face the challenge of being ambiguous and noisy for topic modelling (Albalawi et al. [4]) – we use the text of the lyrics from the 30 seconds selection exclusively. Following Valero et al. [83], we tested different classical and short text topic modelling methods, and evaluated binary classification (FARC-songs or AUC-songs). Thus, in (2) we tested non-negative matrix factorisation (NMF), singular value decomposition (SVD), latent dirichlet allocation (LDA), and collapsed Gibbs sampling for dirichlet multinomial mixture (GSDMM) from Yin and Wang [89]. We performed 5-fold cross-validation and report F1-scores in Table 1. As there is no “ground truth” about the optimal number of topics in topic modelling tasks, Chang et al. [16] suggested focusing on assessments that rely on real-world task performance. Therefore, we ran different tests with different number of topics for each method, and selected the algorithm that offered not only the best topic coherence metrics but also the best classification performance – singular value decomposition and 25 topics. Topic coherence was assessed using the $C_{v}$ metric in Gensim, which estimates how often the most important words of a topic appear together in the text and measures their overall semantic similarity (Röder et al. [65]). Although 25 topics for 100 songs may seem high, this configuration achieved the best coherence and F1 scores and reflected the thematic complexity of the corpus. Many songs contain highly specific references to political, historical, or criminal figures and events in Colombia, which benefit from finer-grained topic resolution; qualitative inspection confirmed that the resulting topics were interpretable and aligned with known narratives. Finally, we obtained a feature matrix that represents the data from the lyrics and which we use as input to a logistic regression classifier: 100 songs with lyrics × 25 topics calculated using SVD.

**Table 1:** Classical and short text topic modelling (TM) approaches tested to produce lyrics models. Bold indicates the highest F1-score and coherence metric. * Indicates the selected model (SVD x 25 topics)

Algorithm	F1-score/Coherence						
	5 topics	10 topics	15 topics	20 topics	25 topics*	30 topics	35 topics
NMF	0.556/0.466	0.588/0.433	0.464/0.398	0.551/0.389	0.628/0.368	0.625/0.458	0.551/0.421
SVD*	**0.711**/0.531	**0.687**/0.592	**0.714**/0.582	**0.709**/**0.650**	**0.723**/**0.647**	**0.675**/**0.697**	**0.777**/0.618
LDA	0.527/0.447	0.428/0.452	0.497/0.413	0.518/0.434	0.469/0.464	0.641/0.514	0.516/0.627
GS-DMM	0.622/0.307	0.642/0.309	0.650/0.325	0.595/0.465	0.610/0.455	0.587/0.495	0.556/0.543

In summary, we produce four types of models: *ACO* models use acoustic features, *LYR* models use features extracted using topic modelling on the lyrics, *MIX* models with use both acoustic and lyrics features, and *RAND* models that pseudo-randomly present music to the participants (i.e., no entropy is calculated).

## Results

A total of 194 participants started the experiment. 52 participants completed the study. Out of these, 3 participants were removed from the completed entries since the server failed to collect their annotations, leaving a total of 49 participants for the analysis. Participants represented a range of age groups ($\mu =35.6$, $\sigma =12.75$): 19 participants were 18-30 years old, 23 participants were 30-50 years old, and 7 participants were 50-65 years old. We had unbalanced participation with respect to gender: 19 female, 29 male, and 1 non-binary. Regarding musical self-identification, most of our participants were non-musicians.^7^ 15 participants received the *MIX* model, 13 received the *RAND* model, 11 received the *LYR* model, and 10 received the *ACO* model. Across the excerpts presented to participants, the distribution of FARC and AUC excerpts was comparable. A t-test confirmed that there was no significant difference between the total number of FARC and AUC excerpts per participant, $t(48) = -0.27$, $p = .788$. Similarly, when restricting the comparison to excerpts with lyrics, no significant differences emerged between FARC and AUC, $t(48) = -0.32$, $p = .754$. In contrast, a significant difference was observed between excerpts with and without lyrics, $t(48) = 15.48$, $p < .001$, indicating that participants consistently encountered more excerpts with lyrics than without. For FARC with lyrics, the number of excerpts per participant ranged from 4 to 16 ($\mu = 10.35$, $\sigma = 2.49$), with the median of 11. For AUC with lyrics, counts ranged from 2 to 19 ($\mu = 10.53$, $\sigma = 3.29$), with the median of 10. The number of excerpt without lyrics were also similar across categories: for FARC ranged from 1 to 11 ($\mu = 4.24$, $\sigma = 2.49$), for AUC ranged from 1 to 10 ($\mu = 4.20$, $\sigma = 2.35$). Overall, the total number of excerpts with no lyrics per participant ranged from 2 to 18 ($\mu = 8.45$, $\sigma = 4.34$), with a median of 9.

### Political scale assessment

**Figure 2:**
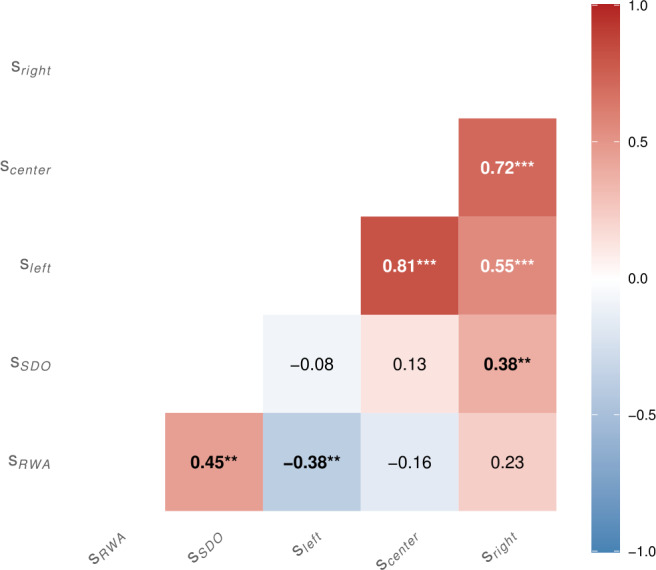
Pearson correlation matrix for political scales. Numbers indicate correlation coefficients (*r*), significant correlations in bold with asterisks denoting significance (p * <.05, **p <.01, ***p <.001)

We obtained five political scores *s* from our three political questionnaires: RWA ($s_{RWA}$), SDO ($s_{SDO}$), PlanLeft ($s_{left}$), PlanCenter ($s_{center}$), and PlanRight ($s_{right}$). For the RWA and SDO, we computed each participant’s score as the mean of their responses to all items in the respective scale (after reverse-scoring where required), resulting in a single score per scale. For the Colombian-specific political questionnaire, we likewise computed the mean agreement score for each candidate’s set of items, yielding three separate scores: $s_{left}$, $s_{center}$, and $s_{right}$. The agreement to the statements of the Colombian specific political questionnaire corresponded to the agreement with the political discourse of a given candidate (i.e., $s_{left}$, $s_{center}$, and $s_{right}$). We computed Pearson correlations between political scales, using pairwise complete observations to assess their effectiveness at capturing political leaning. All five scales correlated, as predicted, showing that the RWA and SDO scores capture left/right polarisation in the Colombian population, presented in Fig. 2. High correlation between $s_{center}$, $s_{left}$ and $s_{right}$, $s_{center}$ could result from the possible lack of knowledge of the candidates’ proposals.

Next, we segmented the participants into three groups: “right-leaning”, “left-leaning”, and “center” orientation. To produce these groups: (1) we grouped participants using the 33% percentile from the RWA and the SDO scores –namely, $s<0.33$ are “left”, $0.33\leq s \leq 0.66$ are “center”, and $s>0.66$ are “right”; (2) we group participants from the Colombian specific questionnaire depending on the highest score obtained – for example, we assign “left” if $s_{left} > s_{right}$ and $s_{left} > s_{center}$, and (3) we obtain the final group by taking the mode from the three resulting classes – for example, if RWA results in “right”, SDO results in “center”, and the Colombian specific questionnaire results in “right”, we assign the participant to the group “right”. Using this score, our participants were grouped as follows: 22 participants are “center”, 15 participants are “left-leaning”, and 12 participants are “right-leaning”.

### Annotation analysis

Gómez-Cañón et al. [32] have argued that inter-rater agreement must be routinely analysed and reported in studies that involve MER, as it quantifies the extent to which listeners make consistent emotional judgments. In Table 2, we summarise inter-rater reliability statistics calculated from the discrete categories used for the MER algorithms: nominal Krippendorff’s coefficient $\alpha _{k}$. To calculate the statistics, we keep only the songs that have been annotated by at least two participants (140 songs from 150 songs) – this reveals that given the initial seed of 6 songs, the response and model variability allowed the participants to annotate most of the data. As mentioned previously, the *RAND* model would produce pseudo-random presentations of songs to be annotated – using the same random seed resulted in 13 participants that annotated the same 30 songs (9 AUC-songs, 12 FARC-songs, and 9 songs without lyrics). Moreover, all participants annotated the same initial 6 songs. We discuss inter-rater reliability and consistency statistics as follows: (1) agreement as measured by $\alpha _{k}$ is notably low in general – we argue that the sparsity of the annotations leads to increasing the probability of agreement due to chance and lowering the agreement coefficient (i.e., each participant annotated 30 from a pool of 150 songs); (2) Cronbach’s *α* ranged from 0.6 to 0.9 for initially rated songs – the initialization of the experiment shows consistent initialization by all participants; (3) annotations of valence are more consistent than annotations of quadrants and arousal for most groups – this is a surprising finding, since typically valence is the most subjective quality and exhibits least consistency; (4) annotations of music without lyrics show a low consistency for “right-leaning” participants – it is likely that the music without lyrics is interpreted with more freedom (e.g., vallenatos and corridos are music normally used for parties); (5) as expected, the response variability of induced emotions from music is evident – using personalised models to capture response variability is a reasonable approach to create MER models (Gómez-Cañón et al. [34], Yang et al. [88]).

**Table 2:** Inter-rater reliability statistics. We report Krippendorff’s $\alpha _{k}$. Q stands for quadrants, A for arousal, and V for valence for each political group

	All Users			Left (n = 15)			Center (n = 22)			Right (n = 12)		
	Q	A	V	Q	A	V	Q	A	V	Q	A	V
All	0.035	0.025	0.051	−0.047	−0.088	−0.067	0.045	0.018	0.131	0.028	0.058	−0.013
Lyrics	0.028	0.025	0.019	−0.033	−0.098	−0.054	0.045	0.040	0.072	0.031	0.032	−0.006
No Lyrics	0.052	0.027	0.122	−0.082	−0.082	−0.093	0.047	−0.033	0.260	0.024	0.133	−0.031

**Figure 3:**
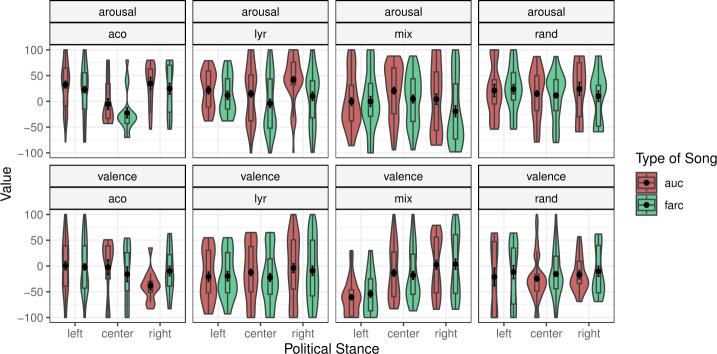
Violin plots with Mean±SE of annnotation analysis for music with lyrics showing the comparisons with respect to model type, annotation (continuous arousal and valence), and political stance

To address R1 and test whether individuals’ political values influence core-affect responses to politically sensitive music, we conducted a Linear Mixed Model (LMM) separately for continuous ratings of arousal and valence. The models included four fixed factors (model [*RAND*, *LYR*, *MIX*, *ACO*], type of music [FARC-songs / AUC-songs], political orientation of the participant [Left / Center / Right], and gender), one interaction effect between political orientation and type of music, and two random factors (participant and track). Comparisons between music with and without lyrics were not assessed because the hypotheses were based around music with lyrics. As such these analyses were run on a subset of the data that only included music with lyrics. The full table of results can be seen in the supplementary material (see section C for music with lyrics, and section D for music with and without lyrics). For valence ratings, main effects for the *MIX* model ($\beta =-30.9$, $t= -2.09$, *p* =.043) and gender ($\beta =24.83$, *t* = 2.36, *p* =.023) emerged. We refer the reader to Fig. 3 for a visualisation of the annotations of music with lyrics. Contrasts in the valence annotations did not show significant differences in the AUC and FARC music across political modes. For arousal ratings, a main effect for gender ($\beta =-16.3$, $t= -2.17$, *p* =.036) and an interaction between the type of music and political orientation [Right] ($\beta =-16.98$, $t=-2.12$, *p* =.035) emerged. Post hoc comparisons of this main effect show that arousal annotations were significantly higher for the AUC songs compared with the FARC songs for right-leaning (*t(945.9)* = 3.1, *p* $< =.002$, *d* =.4) and center-leaning participants (*t(725.5)* = 2.47, *p* =.014, *d* =.25) regardless of the model. Additional comparisons suggest these effects were more pronounced in *LYR* model for both right-leaning participants annotations (*t*(939.8) = 3.03, *p* =.0.003, *d* =.62) and for center-leaning participants (*t*(943.9) = 2.04, *p* =.012, *d* =.42). No differences emerged for left-leaning participants within or between models.

### Algorithmic evaluation

**Figure 4:**
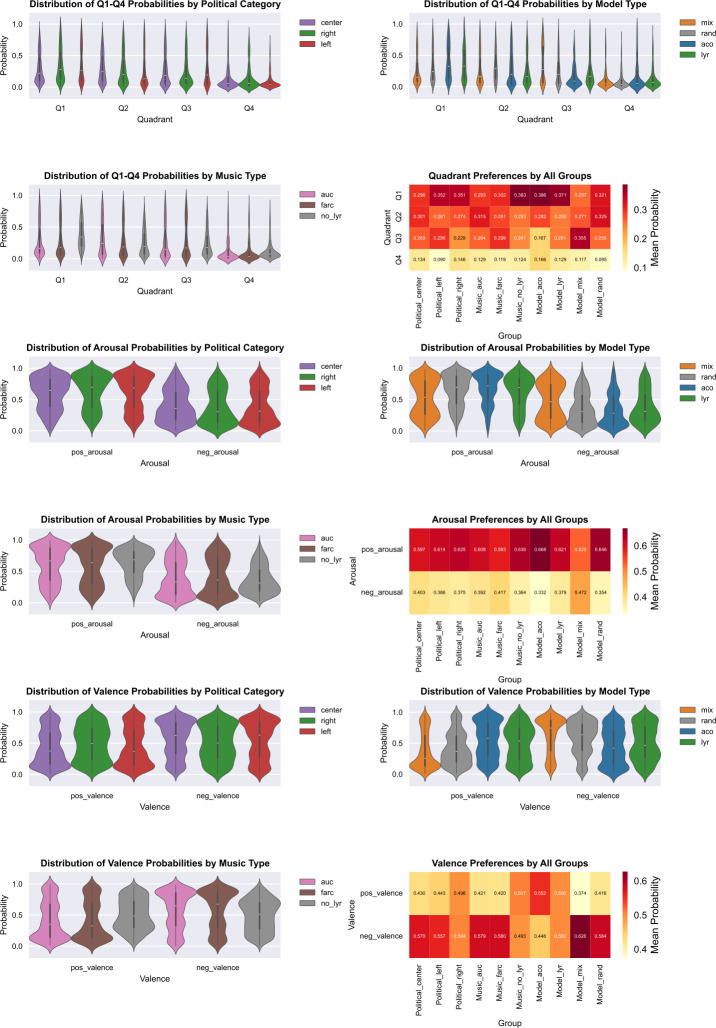
Distribution of personalized models output probabilities across all testing data

To evaluate personalised models, it should be noted that there was *no testing data* – all annotated data were used to train the models and there is no “ground truth” to compare the predictions of the models. However, we propose an evaluation strategy to account for the possible bias that results from “misusing” the personalised models. Given that there is a significant difference in the way that participants from different groups annotated the music, it is likely that the models are inherently biased towards certain categories (Nigam et al. [57], Schölkopf et al. [73]) – moreover, the only evaluation possible is probabilistic given the choice of the algorithms for machine learning. Thus, each personalised model was used to test the remaining data from each participant (see output distributions in Fig. 4), following our previous work (Gómez-Cañón et al. [35]) – the resulting matrix has 120 songs × 4 quadrants. We sorted the matrix according to the highest probabilities of belonging to a particular class and select the top 10 and 20 predictions that: belong to a particular quadrant (i.e., $Q_{1}$, $Q_{2}$, $Q_{3}$, and $Q_{4}$), belong category of arousal (i.e., positive and negative), and belong to a category of valence (i.e., positive and negative). Table 3 summarises the findings that we deem ethically problematic – the type of music that some personalised models appear to classify with high probability of negative valence reveals the political position of the participants. In general, we find that: (1) in the case of “left-leaning” participants, the *LYR*, *MIX*, and *RAND* models predict that AUC-music (i.e., “right-wing”) has a higher probability of inducing negative valence than FARC-music; (2) in the case of “right-leaning” participants, we find that only the *MIX* and *RAND* models predict that FARC music (i.e., “left-wing”) has a higher probability of inducing negative valence than AUC-music; (3) we find no trends as to the type of models that might capture the political stance (i.e., only the *MIX* models do so consistently for the left- and right-leaning groups) – it is likely that the classification strategy might be too coarse to capture the response diversity of each participant and subtle political differences; (5) despite that the political stance from the participants was not necessarily captured by all the models, we find that the models are accurately capturing that *both* types of music might induce emotions with negative valence – this was expected since the political content of the lyrics was strong and specific memories with a negative connotation might have been triggered through music listening; (6) we find that certain participants resulted with models that are the most problematic (i.e., 10 out of 27 personalised models would indeed reflect their political stance) – we offer a summary of predictions in the complementary website for clarity on the behaviour of each participant’s model.^8^

**Table 3:** Proportion of the top 10 and top 20 aggregated predictions from personalised models (in this case, for predictions of negative valence). Bold indicates the highest proportion between AUC, FARC and songs without lyrics

Political leaning	Model type	Top 10			Top 20		
		AUC (%)	FARC (%)	No Lyr. (%)	AUC (%)	FARC (%)	No Lyr. (%)
Center	Acoustic	35.0	65.0	0.0	40.0	60.0	0.0
	Lyrics	42.0	50.0	8.0	48.0	42.0	10.0
	Mix	53.3	35.0	11.7	45.0	40.8	14.2
	Random	34.4	53.3	12.2	38.3	48.9	12.8
Left	Acoustic	43.3	**51.7**	5.0	43.3	**46.7**	10.0
	Lyrics	**65.0**	35.0	0.0	**55.0**	45.0	0.0
	Mix	**50.0**	30.0	20.0	**50.0**	29.0	21.0
	Random	**35.0**	**35.0**	30.0	35.0	27.5	**37.5**
Right	Acoustic	**50.0**	40.0	10.0	42.5	**45.0**	12.5
	Lyrics	**45.0**	22.5	32.5	**38.8**	28.8	32.5
	Mix	25.0	**40.0**	35.0	25.0	**38.8**	36.3
	Random	30.0	**45.0**	25.0	22.5	**40.0**	37.5

We fitted mixed-effects models with participant-level random intercepts to account for the nested structure of the output probabilities ($n=5,643$ output probabilities across 49 participants). Multivariate analysis of variance was used to test the joint significance of predictor effects across the four quadrants. All main effects showed significant multivariate impacts on personalized model predictions: music type (Wilks’ $\lambda = 0.970$, $p < 0.001$), political leaning (Wilks’ $\lambda = 0.967$, $p < 0.001$), and model type (Wilks’ $\lambda = 0.906$, $p < 0.001$). Follow-up univariate tests confirmed systematic effects on the raw quadrant probabilities across all three factors.

**Political orientation.** Political leaning shaped quadrant predictions across all four quadrants. The center-leaning participants had higher $Q_{1}$ probabilities than the left ($diff = 0.057$, $p < 0.001$) and right participants ($diff = 0.055$, $p < 0.001$). For $Q_{2}$, the center-leaning participants differed from the left ($diff = -0.040$, $p < 0.001$) and the right ($diff = -0.027$, $p = 0.003$). $Q_{3}$ probabilities were elevated for the center vs. the right ($diff = -0.040$, $p < 0.001$) and for the left vs. the center ($diff = 0.027$, $p = 0.002$), with the left vs. the right also differing ($diff = -0.067$, $p < 0.001$). Finally, $Q_{4}$ showed differences between the center and the left ($diff = -0.044$, $p < 0.001$) and the left vs. the right ($diff = 0.056$, $p < 0.001$). These results indicate that political orientation systematically influenced the classification patterns of personalized models.

**Model type.** Model architecture had the largest overall multivariate effect, particularly on $Q_{3}$ ($F = 134.036$, $p < 0.001$, $\eta ^{2} = 0.067$) and $Q_{1}$ ($F = 78.016$, $p < 0.001$, $\eta ^{2} = 0.040$). Post-hoc tests revealed systematic differences among all model types. For $Q_{1}$, both the ACO and LYR models produced lower predictions than MIX and RAND. For $Q_{2}$, RAND predictions were higher than ACO, LYR, and MIX. For $Q_{3}$, ACO consistently predicted higher probabilities than LYR, MIX, and RAND, while MIX was lower than LYR and RAND. Finally, $Q_{4}$ predictions for ACO were reduced relative to all other models. Overall, the model architecture strongly shaped personalized quadrant predictions, with ACO favoring mid-valence quadrants and RAND increasing low-valence predictions.

**Interactions.** Significant interactions emerged between music type and political orientation, showing that the effect of music on quadrant probabilities depended on participants’ political leanings. For $Q_{1}$, music without lyrics increased probabilities overall ($\beta = 0.137$, $p < 0.001$), but this effect was reduced for the center- and the right-leaning participants ($\beta = -0.125$, $p < 0.001$). For $Q_{2}$, FARC music increased $Q_{2}$ probabilities for the left-leaning participants ($\beta = 0.037$, $p = 0.012$), whereas music without lyrics increased $Q_{2}$ for right-leaning participants ($\beta = 0.043$, $p = 0.004$). In $Q_{3}$, music without lyrics increased probabilities for right-leaning participants ($\beta = 0.067$, $p < 0.001$), while FARC decreased $Q_{3}$ for both left- ($\beta = -0.040$, $p = 0.004$) and right-leaning participants ($\beta = -0.040$, $p = 0.005$). Finally, in $Q_{4}$, music without lyrics slightly decreased probabilities for left-leaning participants ($\beta = -0.026$, $p = 0.012$), whereas FARC increased $Q_{4}$ for right-leaning participants ($\beta = 0.030$, $p = 0.004$). Overall, the effects of music type on quadrant probabilities were contingent on political orientation. Political orientation interacted strongly with personalization models. Left-leaning participants paired with the *MIX* model exhibited a large reduction in $Q_{1}$ ($\beta = -1.782$, $p = 0.014$), whereas right-leaning participants paired with the *MIX* model experienced a substantial increase in $Q_{4}$ ($\beta = 2.148$, $p = 0.014$). Similarly, left-leaning participants paired with the *RAND* model showed dramatic increases in $Q_{4}$ ($\beta = 2.407$, $p = 0.004$). These political × model interactions were among the strongest observed, amplifying quadrant-specific differences with effect sizes reaching $\eta ^{2} = 0.041$ ($Q_{3}$) for model type. Interactions between model type and music type were generally smaller but consistent. Music without lyrics with the *RAND* model decreased $Q_{1}$ by 1.76 × ($\beta = -0.563$, $p < 0.001$) while increasing $Q_{3}$ by 1.60 × ($\beta = 0.469$, $p < 0.001$). FARC music with the *MIX* model increased $Q_{4}$ ($\beta = 0.285$, $p = 0.0002$), and with the *RAND* model by 1.19 × ($\beta = 0.171$, $p = 0.043$). Music without lyrics with the *LYR* model increased $Q_{4}$ ($\beta = 0.316$, $p = 0.0002$). These patterns indicate that, while model personalization amplifies political differences, it also interacts with musical context depending on the quadrant.

## Discussion

We discuss our findings regarding our proposed hypotheses in the context of the wider research questions as follows:

1. Politically sensitive music stimuli that agrees/disagrees with participants political stance (goal-congruence) will show different induced core affect annotations.
2. Personalised algorithms will be effectively biased towards specific affective quadrants depending on the political stance of listeners – namely, personalised algorithms might reflect the listeners’ political views.

These results partially support H1. That is, a significant main effect for the interaction between political values and the type of music (goal-congruence) was observed in arousal annotations. These findings have direct implications for the design of emotion-aware systems, particularly in politically sensitive contexts where user preferences may encode identity-relevant information. Furthermore, contrasts indicate that arousal annotations by right and center-leaning participants were significantly higher for AUC (right-orientated) music than FARC (left-orientated) music across MER models. This suggests that how well the stimuli align with the participant’s political values influences the induced arousal annotations. The corresponding effect sizes (*d* = .4 for right-leaning; *d* = .25 for center-leaning participants) indicate a small but nontrivial impact, suggesting that goal-congruence has a real-world influence on induced arousal beyond statistical significance. However, the direction of change cannot be interpreted from these results. Congruent music may amplify induced arousal annotations. Alternatively, non-congruent music may suppress induced arousal annotations; both may be true. The inclusion of a control group (e.g., a-political Colombians or non-Colombian nationals) would be required to establish a baseline and subsequently the direction of these effects. Regardless, both interpretations would suggest that emotional responses to music are shaped by socio-political values.

Further comparisons between models allow us to acknowledge that the main effect on arousal was most prevalent in the *LYR* model for both right-leaning participants and center-leaning participants. Notably, the effect sizes in the LYR model were larger (*d* = .62 for right-leaning; *d* = .42 for center-leaning participants), indicating a moderate practical impact and reinforcing that lyrics alone may heighten goal-congruent arousal more than multimodal presentations. This supports our assumption that lyrics play an important role in how a piece of music is appraised as congruent or not and, subsequently, annotated in terms of arousal. Nevertheless, it is worth noting that the use of political values as the operationalization of goals is just one possible variation of the numerous values, associations, and needs individuals apply to music. It may be that different operationalizations of goals may work differently in terms of their effect on core affect or how operationalizations may change the focus one might place on different aspects of a stimulus (Weining [85]). For example, the music or the lyrics. Future work should consider the ethical implications of different operationalizations – particularly in scenarios where model outputs might inform public-facing features such as playlist suggestions, political content moderation, or targeted messaging.

A main effect of valence was also observed in the *MIX* model’s presentation of “negative” stimuli. This shows lower valence annotations in left-leaning participants. However, planned contrasts for valence annotations did not show any interaction with the type of music (goal-congruence), as hypothesised. Political values independent of the degree of congruence with the musical stimuli influenced annotations of valence. This suggests that beyond our initial hypothesis (H1), but in support of our broader research question (R1), political values also independently influence the core affect, at least for valence. Due to the implicit nature of our operationalisation of goal-relevance (the run-up to the Colombian political elections), it is not possible to determine if differences in political values are directly related to valence annotations. The result is nonetheless, tentatively supportive of our wider research questions (R1), while requiring further empirical support through direct manipulation (see Limitations).This trend, from left to right-leaning participants, was only observed in the *MIX* model (see Fig. 3). This points to a critical consideration for multimodal MER systems: the integration of acoustic and lyrical features may increase the granularity of identity-relevant inferences, enhancing predictive power, while simultaneously increasing ethical risk. At the same time, the importance of safeguards – such as context-aware metadata – in the design of emotionally intelligent systems that intersect with socially or politically charged domains.

We have shown that H1 is tentatively supported by these data, as is our main research question (R1). This finding provides empirical evidence that political leanings can influence induced emotional responses to music when listening to politically charged lyrics. The results suggest that the political orientation of the music mediates arousal annotations, demonstrating that personalised emotion recognition systems may implicitly encode users’ political identity. In addition, left-leaning political orientations annotated politically charged music with more negative valence in the *MIX* model. The exploratory nature of these hypotheses suggests a cautious interpretation and the need for replication. However, this leaves an open question for future research to address and poses a possible challenge to existing MER work to better recognise the role of individual, situational, and contextual factors. As such, these findings contribute to the emerging conversation around ethical algorithm design and the need for more inclusive and context-aware affective algorithms (Baeza-Yates [8], Dash and Agres [21], Gómez et al. [31], Kang and Herremans [44]).

In relation to our second hypothesis H2, we expected that personalised models from left and right-leaning participants would reflect that incongruent music would show a high probability of belonging to high arousal and negative valence – polarising lyrics spark different opinions, which in turn result in different annotations and diverse predictions according to the algorithms. To build on our previous study, in this experiment we added several more levels of complexity: we used music without lyrics in the same styles, we included the *LYR*, *MIX*, and *RAND* models, and we allowed our participants to annotate the music using continuous arousal and valence sliders. To some extent, machine learning algorithms are able to capture the political stance of some participants – namely, the model of a “left-leaning” participant would show high probability that AUC-music (“right-wing”) would induce negative valence. However, the political views of the personalised algorithms were not necessarily reflected in each of the models. In general, the broad assumption behind this experiment was that strong political positions should be reflected accordingly with arousal and valence annotations – however, it is likely that participants from different or less extreme political views (e.g., center-left / center-right) would find that both types of music produce emotions with negative valence similarly. We do not find strong support that the models have effectively captured the political stance of all the participants. Tests show that several differences across the personalized models are statistically significant; however, the bias toward reflecting participants’ political stance was not consistent across all models. Nonetheless, 11 out of 27 personalised models from the right/left groups show a high probability that music belonging to an incongruent political stance would induce emotions with negative valence. This provides proof-of-concept that individualised emotion modelling can carry embedded ideological information – a finding with ethical implications for recommender systems. We find it particularly interesting that all models were able to capture that music belonging to both music styles (which are strong emotional stimuli) would induce negative valence. Table 3 shows that most of the models for all groups of participants would predict that music with lyrics would induce negative valence, as opposed to music without lyrics. Thus, the personalization strategy appears to be effective in identifying that music with negative valence is primarily associated with AUC and FARC tracks.

Beyond the initial hypotheses we set out, we have also provided tentative evidence for the utility of the right-wing authoritarian (RWA) scale and the social dominance orientation (SDO) scale in a Colombian population. This is tentative because the sample size is too small to draw conclusions about the population in general. However, they behave and correlate as expected with each other and with our Colombian specific political questionnaire. The SDO and the RWA scales are highly important to our interpretation of the data. A critical look at these scales may shed some light on why the effects were explicitly seen in “right-leaning” participants. In relation to H1 and the valence and arousal annotations, many of the observed effects were noted specifically in “right-leaning” participants. However, the distribution of participants in the SDO and the RWA both produce a left-skew suggesting greater “left-leaning” ideologies (supported by the results of the presidential elections). This may mean that some effects in the “left-leaning” participants are somewhat muted by the “center” grouping.

### Limitations and future directions

We note several limitations in the design of the experiment and some future directions for researchers in music cognition and MER to take.

- Dropout rate: The number of participants who started the experiment but did not complete it was substantially higher than typical for online experiments (Eerola et al. [26], Hoerger [38]). This may have been due to the length of the experiment. It may also have been due to the nature of the experiment, which attempted to allow algorithms to identify music that can induce negative affects in participants. Some participants reported feeling uncomfortable when listening to music of the opposite political view.
- Type of machine learning models: the type of algorithms that were used for the personalisation approach have been established as classical approaches to classification and are efficient models. However, the logistic regression classifier (used in the *ACO*, *LYR*, and *MIX* models) assumes linearity between the acoustic or lyrics features and the annotations from participants – it is likely too coarse to model the subtle non-linearity that relate features to an annotation. Gómez-Cañón et al. [33] showed that faster personalisation could be achieved by the use of convolutional neural network architectures – given the computational requirements of deep learning model estimations, our web servers were not capable of supporting online training.
- Data distributions: although excerpts were drawn from different full songs to prevent data leakage between training and testing, the musical styles from each type (corridos for AUC-music and vallenato/canción social for FARC music) were acoustically similar within each class. This may have limited the selection of acoustically similar excerpts to prior annotations, leading to high confidence in initial predictions and potentially inflating the performance of ACO models.
- Political labels: The political leaning measures (RWA and SDO) showed a predominantly left-skewed distribution. Although this did not substantially affect the analysis – three nearly balanced groups were formed using percentile-based grouping—it is important to note that “right-leaning” participants may be more accurately described as holding “center-right” to “right’ political views. This interpretation is supported by strong correlations between the center, left, and right scores on our Colombia-specific political scale (see Fig. 2). Post-hoc analyses using principal component analysis (PCA) on the raw political scale ratings, followed by K-means clustering into three groups, suggest that the skewness in the scale distributions may have made percentiles a suboptimal grouping method (see supplementary material B). Nonetheless, this alternative approach produced high agreement with our original grouping: 92.3% agreement for the “left” and 66.7% for the “right”. In hindsight, the study could have been strengthened by directly asking participants to self-report their political affiliation. However, self-reports are not without problems: for example, a survey conducted shortly after the elections showed that 71% of Colombians identified as “center,” even though the “left” had just won. This illustrates that even self-reported affiliation can be an unreliable basis for grouping participants. 9
- Musical extremes: It is of course true that these music genres represent quite extreme political ideologies within the Colombian community. The musical genres may be held here to be representative of extreme opinions, although in real life, the connotations are mode nuanced. It is quite possible that they are more representative of far-right/left political ideologies.
- Implicit manipulation: goal-relevance is operationalised implicitly, taking advantage of the run-up to the Colombian political elections. It is not clear, however, if the relevance of the political elections is equal across participants. Moreover, if other mediating factors may influence the relevance of the elections. For example, an individual’s assumption that a particular party might win or lose may change the relevance of the elections. This study did not include such measures due to time constraints. However, controlling for the degree of relevance in future studies would be highly informative.

## Conclusions

This study found evidence that an individual’s political identity contributes meaningfully to their induced emotional experience of music, at least when rated in terms of core affects. Yet, the extent to which political values can influence music-induced emotional episodes remains an open question. Moreover, our findings suggest that algorithmic models have the potential to influence music-induced emotions in real-world scenarios on music with explicit political content, including shifts towards more negative emotional states. We highlight the importance of acknowledging and understanding how MER algorithms with increasing personal data could be used, both in positive and negative scenarios, especially in vulnerable or demographically marginalised groups.

We stress the importance of understanding how emotionally adaptive systems could be used both constructively and harmfully. Although research on music that promotes well-being and beneficial uses has yielded important findings and is likely to grow in the following years (Agres et al. [2], Hu et al. [39]), research on music-induced harm has only begun to be explored (Saarikallio et al. [68], Sharman and Dingle [75], Silverman et al. [77], Surana et al. [81]). It is critical that the field of music technology acknowledges and builds upon the findings of music cognition – there is a necessity to ground technological applications on robust psychological research, since each algorithm will be used to develop specific use cases. Moreover, there is a need to acknowledge that technology poses asymmetrical power relationships onto vulnerable populations (Adams [1], Mohamed et al. [54]) – while the episode from Cambridge Analytica is a well-known situation in Western societies, it is less known that they influenced elections in more than 30 countries and 100 election campaigns (including Colombian elections). This study provides a reflection on the power of music and potential influence on listeners in areas such as politics, strongly linked to emerging risks to democracy.

We conclude by advocating for more socially responsive, context-aware, and ethically guided MER research to address such problems in better understanding the benefits and dangers of emotion annotations in real-world applications of music uses. For researchers in music technology (mainly involved in the MER area), we respectfully suggest the following. Before engaging in implementing the latest machine learning algorithm, assembling huge datasets, or getting state-of-the-art accuracy, we believe that the most relevant question to be addressed is: *what for and at what–or whose–cost?*

## Supplementary Information

Below is the link to the electronic supplementary material.

(PDF 184 kB)

## Data Availability

No datasets were generated or analysed during the current study.

## Footnotes

1 A Colombian folk tradition that translates as “born in the valley”.

2 Core affects are arousal and valence. Arousal refers to energy or activation and valence relates to pleasantness or positiveness of an emotion (Russell [67]).

3 https://trompa-mtg.upf.edu/colombian-not-popular/.

4 Due to the use of a fixed random seed for reproducibility, the system was initialised identically for all participants in this condition. As a result, while the song selection was random in the sense that it did not depend on participants’ prior annotations (unlike in the personalised conditions), all participants in RAND received the same sequence of 30 excerpts..

5 https://www.audiosourcere.com.

6 https://github.com/csteinmetz1/pyloudnorm.

7 27 non-musicians, 6 amateur musicians, 5 serious amateur musicians, 5 professional musicians, 3 semi-professional musicians, and 3 music loving musicians.

8 https://trompa-mtg.upf.edu/colombian-not-popular/visualization/

9 https://www.moe.org.co/wp-content/uploads/2024/07/Cuarto-Estudio-de-Opinion-sobre-el-Congreso-de-la-Republica-2022-2026-y-Opiniones-Ciudadanas-1-1.pdf.

## Abbreviations

AI: Artificial Intelligence
MER: Music Emotion Recognition
FARC: Fuerzas Armadas Revolucionarias de Colombia
AUC: Autodefensas Unidas de Colombia
GDPR: General Data Protection Regulation
ITU: International Telecommunication Union
ACO: Acoustic model
LYR: Lyrics model
MIX: Hybrid model
SDO: Social Dominance Orientation scale
RWA: Right-wing Authoritarianism scale
DEAM: Database for Emotional Analysis of Music
TM: Topic modeling
NMF: Non-negative matrix factorization
SVD: Singular value decomposition
LDA: Latent dirichlet allocation
GSDMM: Collapsed Gibbs sampling for dirichlet multinomial mixture
