# Supplementary material for: Personalisation and profiling using algorithms and not-so-popular Colombian music: goal-directed mechanisms in music emotion recognition
Source: EPJ Data Sci. 2025 Nov 13;14(1):80. doi: 10.1140/epjds/s13688-025-00595-1 (PMC12615516; doi:10.1140/epjds/s13688-025-00595-1)
Supplement: Supplementary file 1 — (PDF 184 kB) [file 13688_2025_595_MOESM1_ESM.pdf]

Supplementary Material

A Political Scales

Right-wing authoritarianism (RWA) scale questions. Measured on a 5-point likert scale; 1 = 'strongly disagree' and 5 = 'strongly agree'.

1. Our country needs a powerful leader, in order to destroy the radical and immoral currents prevailing in society today.
2. Our country needs free thinkers, who will have the courage to stand up against traditional ways, even if this upsets many people.
3. The "old-fashioned ways" and "old-fashioned values" still show the best way to live.
4. Our society would be better off if we showed tolerance and understanding for untraditional values and opinions.
5. God's laws about abortion, pornography and marriage must be strictly followed before it is too late, violations must be punished.
6. The society needs to show openness towards people thinking differently, rather than a strong leader, the world is not particularly evil or dangerous.
7. It would be best if newspapers were censored so that people would not be able to get hold of destructive and disgusting material.
8. Many good people challenge the state, criticize the church and ignore "the normal way of living".
9. Our forefathers ought to be honored more for the way they have built our society, at the same time we ought to put an end to those forces destroying it.
10. People ought to put less attention to the Bible and religion, instead they ought to develop their own moral standards.
11. There are many radical, immoral people trying to ruin things; the society ought to stop them.
12. It is better to accept bad literature than to censor it.
13. Facts show that we have to be harder against crime and sexual immorality, in order to uphold law and order.
14. The situation in the society of today would be improved if troublemakers were treated with reason and humanity.
15. If the society so wants, it is the duty of every true citizen to help eliminate the evil that poisons our country from within.

Social dominance orientation (SDO) scale. Measured on a 5-point likert scale; 1 = 'strongly oppose' and 5 = 'strongly favor'.

1. An ideal society requires some groups to be on top and others to be on the bottom.
2. Some groups of people are simply inferior to other groups.
3. No one group should dominate in society.
4. Groups at the bottom are just as deserving as groups at the top.
5. Group equality should not be our primary goal.
6. It is unjust to try to make groups equal.
7. We should do what we can to equalize conditions for different groups.
8. We should work to give all groups an equal chance to succeed.

Colombian specific political questionnaire. Measured on a 5-point likert scale; 1 = 'strongly oppose' and 5 = 'strongly favor'.

1. We have to combat criminal organization by increasing the operative capabilities and presence of the public force.
2. We must develop a program of citizen cohabitation as a mechanism to prevent crime and solve conflicts.
3. The reasons for insecurity are: hunger, non-inclusion of young citizens, and the lack of compliance with the peace treaty.
4. We must strongly fight FARC dissidents that cheated us by taking advantage of the peace treaty to become strong and continue committing crimes.
5. We should qualify the ESMAD with classes on human rights, political and social aspects of the national reality.
6. It is necessary to dismantle the ESMAD and to transit to a new force which is oriented to peaceful and intelligent solution of conflicts.
7. We should generate conditions for greater investment in responsible mining/energy projects.
8. The transition between coal mining and oil to renewable energies is necessary, but should be accomplished gradually.
9. We should start the transition to clean and renewable energies now, leaving aside the extractivist logic of the oil industry.

B Distribution of political scales

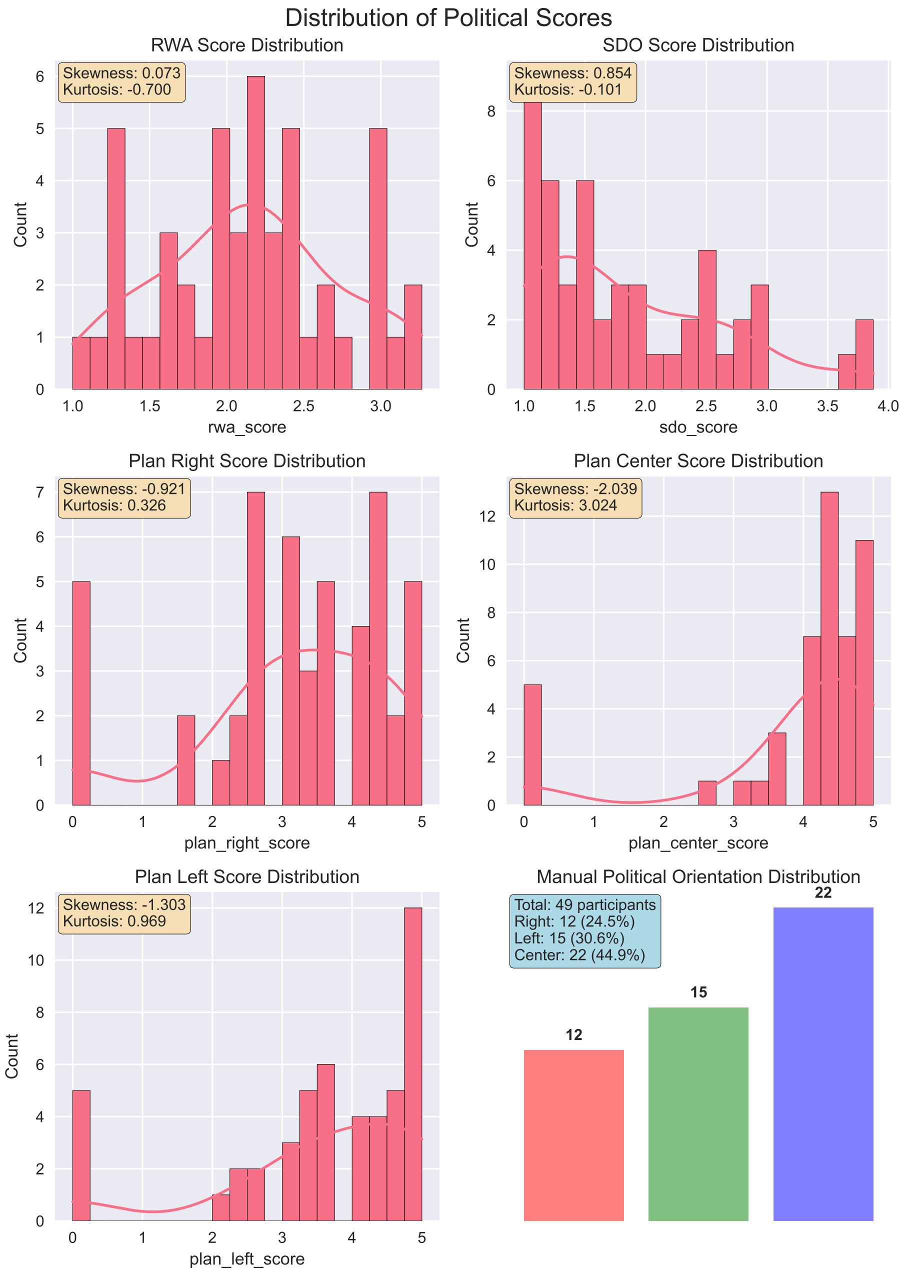

Figure 1: Distribution of the political scales and manual classification.

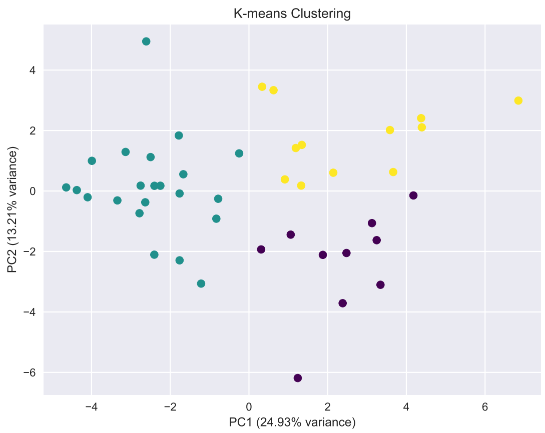

Figure 2: Principal component analysis from the raw results and K-means clustering.

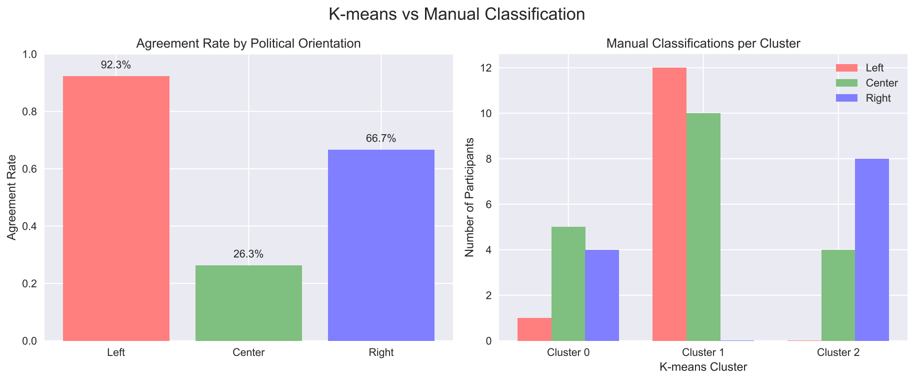

Figure 3: Comparison of k-means clustering and manual classification using 33/66% percentiles.

C Linear Mixed Model Comparisons for music with Lyrics

C.1 Valence Linear Mixed Models

Table 1: Linear mixed model of valence annotations for music with lyrics

	Estimate	Std. Error	df	t value	p.value
(Intercept)	-23.343	13.294	45.944	-1.756	0.086
modeLyrics	-12.233	16.019	40.749	-0.764	0.449
modeMixed	-30.886	14.759	41.628	-2.093	0.043
modeRandom	-16.902	15.675	41.749	-1.078	0.287
typefarc	0.615	4.956	955.548	0.124	0.901
political_modeCenter	9.778	12.931	46.006	0.756	0.453
political_modeRight	17.755	14.235	46.761	1.247	0.219
gendermale	24.829	10.522	40.618	2.360	0.023
typefarc:political_modeCenter	-1.517	6.278	946.033	-0.242	0.809
typefarc:political_modeRight	2.419	7.055	941.107	0.343	0.732

C.2 Arousal Linear Mixed Models

Table 2: Linear mixed model of arousal annotations for music with lyrics

	Estimate	Std. Error	df	t value	p.value
(Intercept)	30.761	9.849	51.505	3.123	0.003
modeLyrics	0.921	11.472	40.060	0.080	0.936
modeMixed	-13.030	10.654	42.213	-1.223	0.228
modeRandom	-3.031	11.286	41.894	-0.269	0.790
typefarc	-1.457	5.590	950.266	-0.261	0.794
political_modeCenter	0.997	9.668	53.483	0.103	0.918
political_modeRight	5.528	10.701	55.453	0.517	0.608
gendermale	-16.301	7.531	39.812	-2.165	0.036
typefarc:political_modeCenter	-10.227	7.129	960.793	-1.435	0.152
typefarc:political_modeRight	-16.975	8.024	954.726	-2.115	0.035

Table 3: Arousal annotations pairwise contrasts for type of music by political orientation for music with lyrics

contrast	political_mode	estimate	SE	df	t.ratio	p.value
auc - farc	Left	1.099643	5.609361	952.3755	0.1960372	0.8446229
auc - farc	Center	11.575773	4.679978	725.5400	2.4734672	0.0136088
auc - farc	Right	18.335281	5.917839	945.8646	3.0983070	0.0020038

Table 4: Arousal annotations pairwise contrasts across models and political orientation

contrast	political_mode	mode	estimate	SE	df	t.ratio	p.value
auc - farc	Left	Acoustic	2.1833216	9.013046	954.7257	0.2422401	0.8086461
auc - farc	Center	Acoustic	23.2735144	16.340171	953.8938	1.4243128	0.1546830
auc - farc	Right	Acoustic	-0.4505535	16.544023	951.3976	-0.0272336	0.9782791
auc - farc	Left	Lyrics	7.9771982	13.043318	933.7443	0.6115927	0.5409561
auc - farc	Center	Lyrics	19.3559854	9.486501	943.8713	2.0403713	0.0415912
auc - farc	Right	Lyrics	28.5359318	9.431207	939.7907	3.0256924	0.0025482
auc - farc	Left	Mixed	-0.0536915	10.461402	948.9655	-0.0051323	0.9959061
auc - farc	Center	Mixed	15.1620813	8.970414	945.8114	1.6902320	0.0913131
auc - farc	Right	Mixed	16.7389491	10.373929	943.6494	1.6135592	0.1069574
auc - farc	Left	Random	-4.0539043	14.565977	934.9248	-0.2783132	0.7808335
auc - farc	Center	Random	1.9939352	7.183104	509.4840	0.2775868	0.7814421
auc - farc	Right	Random	12.6266513	14.565977	934.9248	0.8668592	0.3862416

D Linear Mixed Model Comparisons for music with and without lyrics

D.1 Valence Linear Mixed Models

Table 5: Linear mixed model of valence annotations for music with and without lyrics

	Estimate	Std. Error	df	t value	p.value
(Intercept)	-21.273	12.586	44.935	-1.690	0.098
modeLyrics	-12.830	15.291	41.171	-0.839	0.406
modeMixed	-31.482	13.997	40.984	-2.249	0.030
modeRandom	-19.695	14.880	41.258	-1.324	0.193
typefarc	1.553	4.029	1361.505	0.385	0.700
political_modeCenter	10.784	12.224	44.738	0.882	0.382
political_modeRight	17.638	13.457	45.494	1.311	0.197
gendermale	23.730	10.039	40.957	2.364	0.023
typefarc:political_modeCenter	-1.246	5.189	1352.056	-0.240	0.810
typefarc:political_modeRight	2.149	5.943	1343.276	0.362	0.718

D.2 Arousal Linear Mixed Models

Table 6: Linear mixed model of arousal annotations for music with and without lyrics

	Estimate	Std. Error	df	t value	p.value
(Intercept)	33.846	9.439	47.782	3.586	0.001
modeLyrics	-0.240	11.308	41.430	-0.021	0.983
modeMixed	-13.354	10.338	41.029	-1.292	0.204
modeRandom	-7.602	10.987	41.256	-0.692	0.493
typefarc	-2.194	4.434	1327.398	-0.495	0.621
political_modeCenter	-2.420	9.271	49.765	-0.261	0.795
political_modeRight	3.487	10.258	51.631	0.340	0.735
gendermale	-15.027	7.416	41.031	-2.026	0.049
typefarc:political_modeCenter	-8.369	5.755	1365.938	-1.454	0.146
typefarc:political_modeRight	-17.291	6.610	1354.710	-2.616	0.009

Table 7: Arousal annotations pairwise contrasts for type of music by political orientation for music with and without lyrics

contrast	political_mode	estimate	SE	df	t.ratio	p.value
auc - farc	Left	2.031834	4.446437	1329.481	0.4569579	0.647776
auc - farc	Center	10.550323	3.835478	1078.036	2.7507193	0.006046
auc - farc	Right	19.448222	5.011159	1352.901	3.8809825	0.000109

Table 8: Arousal annotations pairwise contrasts across models and political orientation for music with and without lyrics

contrast	political_mode	mode	estimate	SE	df	t.ratio	p.value
auc - farc	Left	Acoustic	-0.3674285	6.926339	1377.1059	-0.0530480	0.9577013
auc - farc	Center	Acoustic	21.3421873	12.080359	1380.4637	1.7666848	0.0775020
auc - farc	Right	Acoustic	9.8398870	12.269877	1377.7649	0.8019548	0.4227174
auc - farc	Left	Lyrics	12.4628457	12.265486	1369.6777	1.0160906	0.3097657
auc - farc	Center	Lyrics	19.8966810	7.954670	1380.9943	2.5012579	0.0124903
auc - farc	Right	Lyrics	24.0767298	8.850628	1377.9258	2.7203415	0.0066035
auc - farc	Left	Mixed	2.5187384	7.619956	1362.4285	0.3305450	0.7410390
auc - farc	Center	Mixed	13.3072169	7.013474	1374.4447	1.8973788	0.0579874
auc - farc	Right	Mixed	26.7827449	8.399619	1380.4373	3.1885668	0.0014619
auc - farc	Left	Random	0.8268271	11.776005	1375.7727	0.0702129	0.9440344
auc - farc	Center	Random	1.6527199	5.706937	982.2027	0.2895984	0.7721846
auc - farc	Right	Random	6.5411128	11.776005	1375.7727	0.5554611	0.5786696
